# Hyperglycemia and kidney outcomes in critically ill children and young adults on continuous kidney replacement therapy

**DOI:** 10.1007/s00467-025-06777-3

**Published:** 2025-04-24

**Authors:** Shrea Goswami, Katja M. Gist, Petter Bjornstad, Eileen Ciccia, Akash Deep, Ben Gelbart, Shina Menon, Eleonora Marinari, Nicholas J. Ollberding, Dua Qutob, JangDong Seo, Danielle E. Soranno, Brynna Van Wyk, Michelle C. Starr, on behalf of the Worldwide Exploration of Renal Replacement Outcomes Collaborative in Kidney Disease (WE-ROCK) Collaborative, Emily Ahern, Ayse Akcan Arikan, Issa Alhamoud, Rashid Alobaidi, Pilar Anton-Martin, Shanthi S. Balani, Matthew Barhight, Abby Basalely, Amee M. Bigelow, Gabriella Bottari, Andrea Cappoli, Abhishek Chakraborty, Eileen A. Ciccia, Michaela Collins, Denise Colosimo, Gerard Cortina, Mihaela A. Damian, Sara De la Mata Navazo, Gabrielle DeAbreu, Akash Deep, Kathy L. Ding, Kristin J. Dolan, Lama Elbahlawan, Sarah N. Fernandez Lafever, Dana Y. Fuhrman, Ben Gelbart, Katja M. Gist, Stephen M. Gorga, Francesco Guzzi, Isabella Guzzo, Taiki Haga, Elizabeth Harvey, Denise C. Hasson, Taylor Hill-Horowitz, Haleigh Inthavong, Catherine Joseph, Ahmad Kaddourah, Aadil Kakajiwala, Aaron D. Kessel, Sarah Korn, Kelli A. Krallman, David M. Kwiatkowski, Jasmine Lee, Laurance Lequier, Tina Madani Kia, Kenneth E. Mah, Eleonora Marinari, Susan D. Martin, Shina Menon, Tahagod H. Mohamed, Catherine Morgan, Theresa A. Mottes, Melissa A. Muff-Luett, Siva Namachivayam, Tara M. Neumayr, Jennifer Nhan, Abigail O’Rourke, Nicholas J. Ollberding, Matthew G. Pinto, Dua Qutob, Valeria Raggi, Stephanie Reynaud, Zaccaria Ricci, Zachary A. Rumlow, María J. Santiago Lozano, Emily See, David T. Selewski, Carmela Serpe, Alyssa Serratore, Ananya Shah, Weiwen V. Shih, H. Stella Shin, Cara L. Slagle, Sonia Solomon, Danielle E. Soranno, Rachana Srivastava, Natalja L. Stanski, Michelle C. Starr, Erin K. Stenson, Amy E. Strong, Susan A. Taylor, Sameer V. Thadani, Amanda M. Uber, Brynna Van Wyk, Tennille N. Webb, Huaiyu Zang, Emily E. Zangla, Michael Zappitelli

**Affiliations:** 1 Division of Nephrology, Department of Pediatrics, Indiana University School of Medicine, Indianapolis, IN USA; 2 Department of Pediatrics, Colorado Children’s Hospital, Aurora, CO USA; 3 Division of Endocrinology, Department of Pediatrics, and Division of Metabolism, Endocrinology and Nutrition, Department of Medicine, University of Washington School of Medicine, Seattle, WA USA; 4 Washington University School of Medicine, St. Louis Children’s Hospital, St. Louis, MO USA; 5 King’s College Hospital, London, England; 6 Royal Children’s Hospital, University of Melbourne, Murdoch Children’s Research Institute, Melbourne, VIC Australia; 7 Division of Nephrology, Department of Pediatrics, Stanford University School of Medicine, Palo Alto, CA USA; 8 Bambino Gesù Children Hospital, IRCCS, Rome, Italy; 9 Sidra Medicine and Weil Cornel Medicine, Doha, Qatar; 10 Weldon School of Bioengineering, Purdue University, West Lafayette, IN USA; 11 University of Iowa Stead Family Children’s Hospital, Carver College of Medicine, Iowa City, IA USA; 12 Division of Child Health Service Research, Department of Pediatrics, Indiana University School of Medicine, 410 W 10 th Street, Suite 2000 A, Indianapolis, IN 46202 USA

**Keywords:** WE-ROCK, Citrate, Glucose, Acute kidney injury, Dialysis, Continuous kidney replacement therapy

## Abstract

**Background:**

There are limited studies evaluating hyperglycemia in children treated with continuous kidney replacement therapy (CKRT). We evaluated the association of hyperglycemia with kidney outcomes in critically ill children treated with CKRT for acute kidney injury (AKI) or fluid overload.

**Methods:**

Secondary analysis of the multicenter retrospective observational Worldwide Exploration of Renal Replacement Outcomes Collaborative in Kidney Disease (WE-ROCK) study (34 centers, 9 countries). Primary exposure was hyperglycemia on days 0–7 of CKRT (average serum glucose of ≥ 150 mg/dL). Average serum glucose < 150 mg/dL was defined as euglycemic. We stratified the hyperglycemic group with cut-offs ≥ 180 mg/dL, ≥ 200 mg/dL, or ≥ 250 mg/dL. The primary outcome was MAKE-90 (death by 90 days or persistent kidney dysfunction [> 125% baseline serum creatinine, or dialysis dependence]).

**Results:**

Of 985 participants, 48% (473) had average serum glucose > 150 mg/dL during days 0–7 of CKRT. There were higher rates of death in the hyperglycemic group (44% vs. 32%, *p* < 0.001) and longer length of stay among survivors (42 vs. 38 days, *p* = 0.017) compared to the euglycemic group. Those with average glucose ≥ 150 mg/dL had higher unadjusted odds of MAKE-90 (OR: 1.36, 95% CI 1.02–1.81); this finding did not remain after multivariate adjustment. Those with average glucose ≥ 180 mg/dL had higher adjusted odds of MAKE-90 (aOR: 1.44, 95% CI 1.02–2.04). In adjusted analysis, each 10 mg/dL increase in glucose was associated with 3% increased odds of MAKE-90.

**Conclusions:**

Hyperglycemia is associated with worse kidney outcomes among young persons on CKRT for AKI or fluid overload. Further studies are needed to evaluate the causality and determine appropriate glucose ranges in this high-risk population.

**Graphical abstract:**

**Figure:**
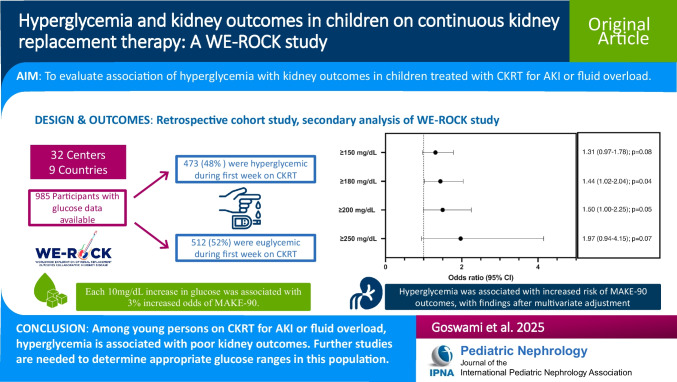
A higher resolution version of the Graphical abstract is available as Supplementary information

**Supplementary Information:**

The online version contains supplementary material available at 10.1007/s00467-025-06777-3.

## Introduction

Hyperglycemia is common in critically ill children and adults [1–3] as is acute kidney injury (AKI) [4]. There is a bidirectional relationship between the pathogenesis of altered glucose metabolism and AKI, as aberrant glucose metabolism contributes to the amplification of inflammatory response during AKI, while AKI leads to loss of kidney glucose homeostasis [5, 6]. Clinical studies examining the relationship between hyperglycemia and AKI in children are limited, with one study reporting higher odds of AKI [7]. Management of hyperglycemia in critical illness and its effect on mortality and other outcomes has been controversial, with conflicting studies in both children and adults [6]. The Kidney Disease Improving Global Outcomes (KDIGO) AKI care bundle highlights the importance of maintaining euglycemia in the care of patients with AKI; however, pediatric-specific data are lacking [8].

Large pediatric trials have evaluated glycemic control in critical illness and have included kidney replacement therapy (KRT) as a secondary outcome with conflicting conclusions. The HALF-PINT (Heart and Lung Failure Pediatric Insulin Titration) Trial, the largest study in critically ill children [9], found no difference in mortality between groups based on glycemic targets, however, they did report that the lower glucose target group had higher rates of kidney replacement therapy (KRT) compared to their higher target glucose group. Contrary to this finding, a recent pediatric meta-analysis concluded that tight glucose control was associated with a decrease in odds of KRT [10].

Despite these studies, much remains unknown about the relationship between hyperglycemia and kidney and systemic outcomes in children and young adults treated with KRT. To our knowledge, there are no studies evaluating the association of glucose control with kidney outcomes and mortality in critically ill children treated with continuous KRT (CKRT). Given these knowledge gaps, we sought to evaluate the association between hyperglycemia and Major Adverse Kidney Events at 90 days (MAKE- 90) in a large, multinational, modern cohort of critically ill children and young adults treated with CKRT for AKI and/or fluid overload (FO). We hypothesized that children and young adults with hyperglycemia would have worse kidney outcomes compared to those with euglycemia.

## Methods

### Study population

This was a planned secondary analysis using data from the Worldwide Exploration of Renal Replacement Outcomes Collaborative in Kidney Diseases (WE-ROCK), a multicenter (34 sites) multinational (9 countries) investigator-driven collaborative. We included infants, children, and young adults < 25 years, receiving CKRT for AKI and FO from January 2015 to December 2021. The study design and overall outcomes have been previously reported [11]. Patients concurrently treated with extracorporeal membrane oxygenation, those with previous dialysis dependence, and those needing CKRT for another indication were excluded. Given the WE-ROCK study design, daily data, including glucose values and insulin use, were only available on CKRT days 0–7. The Institutional Review Board (IRB) at Cincinnati Children’s Hospital Medical Center approved this collaborative study (IRB#2021–0265). Each participating collaborative center received approval with waiver of informed consent from their Human Research Ethics or IRB Committee. This study follows the Strengthening the Reporting of Observational Studies in Epidemiology reporting guidelines (Supplementary Appendix).

### Demographic data

As has been previously reported, demographic data were collected including sex, age at ICU admission, time from ICU admission to CKRT initiation (days), race and ethnicity, weight, and height. Body mass index (BMI) was calculated for children > 2 years old using admission or dry weight and sex-specific BMI-for-age percentiles and subjects were categorized as underweight (< 5^th^ percentile), healthy weight (5^th^ to < 85^th^ percentile), overweight (85^th^ to 95^th^ percentile) and obese (> 95^th^ percentile) [12]. The presence of sepsis was defined as a documented infection and systemic inflammatory response syndrome criteria within 24 h of admission to the ICU [13]. Data at CKRT initiation included serum creatinine (SCr), Pediatric Logistic Organ Dysfunction 2 (PELOD- 2) score, fluid balance, and loop diuretic use [13–16]. Information on anticoagulation usage was reported at the time of CKRT initiation, as citrate directly impacts metabolism by its involvement in the Krebs cycle, the regulatory mechanism of glycolysis, proteolysis, and lipolysis, and alters insulin resistance and satiety signals in the hypothalamus [17]. Baseline SCr was defined as the lowest SCr (mg/dL) within the 3 months prior to admission. If baseline SCr was unknown, a value was imputed using an estimated glomerular filtration rate (eGFR) of 100 mL/min/1.73 m^2^ and body surface area, as previously validated [18].

### Glucose exposure

The highest daily serum glucose and insulin use from days 0–7 of CKRT was collected. Up to 8 daily serum glucose values were available per patient (e.g., days 0–7), but fewer in those with shorter durations of CKRT. Participants were described as hyperglycemic if their average serum (mean) glucose during days 0–7 on CKRT was > 150 mg/dL. The threshold for hyperglycemia is from the PODIUM (Pediatric Organ Dysfunction Information Update Mandate) consensus [19]. As the PODIUM was not developed specifically for young persons receiving CKRT, we also performed a secondary analysis evaluating higher glucose thresholds (≥ 180 mg/dL, ≥ 200 mg/dL, or ≥ 250 mg/dL). Further, we performed additional analysis with glucose as a continuous exposure variable. Additionally, we calculated days in range (DIR) which was defined as percent of days each subject’s peak glucose was euglycemic (< 150 mg/dL). An example of DIR calculation is as below.

**Table:** 

CKRT day	0	1	2	3	4	5	6	7
Daily glucose peak (mg/dL)	130	130	120	154	168	110	120	134
DIR	1	1	1	0	0	1	1	1

DIR: 6/8 = 0.75 or 75% time spent in range

### Outcome

The primary outcome was MAKE- 90, defined as a composite of (1) death, (2) KRT dependence, or (3) persistent kidney dysfunction (> 125% above baseline serum Scr) [11] at 90 days after CKRT initiation. Our secondary outcomes included MAKE- 30, defined as (1) death, (2) KRT dependence, or (3) persistent kidney dysfunction (> 125% above baseline serum Scr) at 30 days, hospital length of stay (LOS) for survivors, and in-hospital mortality.

### Statistical analysis

Data were summarized as median (interquartile range [IQR]) for continuous and frequencies (%) for categorical variables. Categorical variables were compared using Chi-square or Fishers’ exact tests and continuous variables using Wilcoxon rank sum test, as appropriate. Comparisons were made between euglycemia and hyperglycemia to assess for differences, including demographics, clinical characteristics, and MAKE- 90. Multivariable logistic mixed effects regression was used to assess the association between each of the glucose thresholds and MAKE- 90, adjusting for age, PELOD score prior to initiation, presence of sepsis, insulin and citrate use as well as comorbid conditions. Models included random intercepts to account for the clustering of patients within centers. Two-sided *p*-values < 0.05 were considered statistically significant. All analyses were performed using R version 4.3.2 software (R Foundation for Statistical Computing, Vienna, Austria) including RStudio (RStudio Team, 2024), the lme4 package (v1.1–26), and the tidyverse package [20].

## Results

### Patient characteristics

Of the 996 patients in the WE-ROCK registry, 7 were excluded for incomplete glucose data and 4 for pre-existing diabetes mellitus (3 had type 2 diabetes mellitus and 1 had type 1 diabetes mellitus), leaving 985 participants included in this secondary analysis (Supplemental Fig. 1). Among these 985 participants, 473 (48%) were hyperglycemic during the first week of CKRT (Supplemental Fig. 2). Female sex was more common in the hyperglycemic group compared to those with euglycemia (49% vs. 43%, *p* = 0.042). The hyperglycemic group had more participants in the 5–21-year age group and weighed more than the euglycemic group (34 kg [IQR 14, 60] vs. 20 kg [IQR 10, 51], *p* < 0.001). There was no difference in BMI between groups (Table 1).

**Table 1:** Demographic and CKRT characteristics of cohort, comparing euglycemic to hyperglycemic

Characteristic	Euglycemic, *N* = 512^1^	Hyperglycemia *N* = 473^1^	*p*-value^2^
Female sex	218 (43%)	232 (49%)	0.042
Age categories			< 0.001
< 1 month	37 (7.2%)	13 (2.7%)	
1 month–1 year	80 (16%)	48 (10%)	
1–5 years	130 (25%)	96 (20%)	
5–15 years	161 (31%)	175 (37%)	
15–21 years	93 (18%)	123 (26%)	
> 21 years	11 (2.1%)	20 (4.2%)	
Weight	20 (10, 51)	34 (14, 60)	< 0.001
Body mass index^3^			0.425
Underweight	29 (9.1%)	27 (8.0%)	
Healthy weight	172 (54%)	165 (49%)	
Overweight	38 (12%)	48 (14%)	
Obese	80 (25%)	99 (29%)	
Race			0.137
Asian/Pacific Islander	40 (8.8%)	22 (5.3%)	
Black	57 (13%)	66 (16%)	
More than one race	7 (1.5%)	10 (2.4%)	
Native Americans	10 (2.2%)	6 (1.4%)	
White	341 (75%)	311 (75%)	
Ethnicity: Hispanic/Latino	79 (18%)	77 (18%)	0.865
Admission category			0.004
CNS dysfunction	20 (3.9%)	18 (3.8%)	
Other	132 (26%)	77 (16%)	
Pain/sedation management	5 (1.0%)	3 (0.6%)	
Post-surgical/minor trauma	20 (3.9%)	29 (6.1%)	
Primary cardiac: post-surgical	24 (4.7%)	24 (5.0%)	
Congenital heart disease	21 (4.1%)	10 (2.1%)	
Heart failure, cardiomyopathy	22 (4.3%)	18 (3.8%)	
Respiratory failure	82 (16%)	109 (23%)	
Shock/infection/major trauma	186 (36%)	185 (39%)	
Comorbidities			
None	121 (24%)	79 (17%)	0.007
Respiratory	68 (13%)	64 (14%)	0.909
Cardiology	97 (19%)	92 (19%)	0.841
Neurology	72 (14%)	59 (12%)	0.463
Nephrology	43 (8.4%)	46 (9.7%)	0.468
Hematology	73 (14%)	57 (12%)	0.307
Oncology	107 (21%)	124 (26%)	0.049
Immunology	64 (13%)	88 (19%)	0.008
Gastroenterology	74 (14%)	104 (22%)	0.002
Endocrine	19 (3.7%)	40 (8.5%)	0.002
Sepsis	210 (41%)	247 (52%)	< 0.001
PRISM III	14 (10, 19)	14 (9, 18)	0.068
Baseline SCr	0.40 (0.28, 0.63)	0.45 (0.26, 0.66)	0.534
eGFR at ICU admission	45 (21, 90)	58 (30, 92)	0.005
eGFR prior to CKRT initiation	25 (14, 47)	27 (17, 47)	0.121
VIS prior to CKRT	5 (0, 20)	5 (0, 20)	0.583
PELOD prior to CKRT	5.0 (2.5, 8.0)	6.0 (2.0, 8.0)	0.963
Fluid overload categories			0.982
< 10%	294 (58%)	271 (57%)	
10–20%	96 (19%)	90 (19%)	
> = 20%	117 (23%)	111 (24%)	
Anticoagulation use			< 0.001
Citrate	250 (49%)	354 (75%)	
Others	262 (51%)	118 (25%)	
CKRT dose per 1.73 m^2^	2,261 (1,853, 3,009)	2,111 (1,839, 2,697)	0.035
CKRT dose per kg	49 (35, 71)	41 (31, 55)	< 0.001
CKRT dose per kg			0.001
< 25 ml/kg/h	36 (7.3%)	45 (9.6%)	
25–40 ml/kg/h	139 (28%)	176 (37%)	
> 40 ml/kg/h	321 (65%)	249 (53%)	
DIR (Days spent in range)	100 (75, 100)	20 (0, 38)	< 0.001
Insulin used	35 (6.8%)	113 (24%)	< 0.001
Highest insulin rate (7 days)	0.08 (0.05, 0.15)	0.10 (0.05, 0.20)	0.079

^1^*n*/*N* (%); median (IQR), ^2^Pearson’s Chi-squared test, Wilcoxon rank sum test, ^3^body mass index Categories determined using Centers for Disease Control for children aged > 2 years old using normative data and categorized as underweight (< 5^th^ percentile), healthy weight (5^th^ to < 85^th^ percentile), overweight (85^th^ to 95^th^ percentile) and obese (> 95^th^ percentile), ^4^CKRT dose reported is delivered dose and is calculated in those receiving non-SCUF (*N* = 966)

*CKRT*, continuous kidney replacement therapy; *CNS*, central nervous system; *eGFR*, estimated glomerular filtration rate; *ICU*, intensive care unit; *IQR*, interquartile range; *PELOD*, pediatric logistic organ dysfunction; *PRISM*, pediatric risk of mortality; *SCr*, serum creatinine; *VIS*, vasoactive-inotropic score

Shock/infection/trauma was the most common reason for admission in both groups with multiple comorbidities reported in both groups. Oncologic, immunologic, gastroenterology, and endocrine co-morbidities were more common in those with hyperglycemia**.** Sepsis was also more common in those with hyperglycemia compared to those with euglycemia (52% vs. 41%, *p* < 0.001) (Table 1).

Participants in the hyperglycemia group spent a median of 20% (IQR 0, 38) of days in range during the first 8 days of CKRT compared to a median of 100% (IQR 75, 100) of days in range during the first 8 days of CKRT for the euglycemic group (*p* = 0.0001). One-quarter of the patients in the hyperglycemic group were treated with insulin (24.0%) compared to only 6.8% in the euglycemic group (*p* < 0.001). Citrate anticoagulation was more commonly used in those in the hyperglycemic group vs. euglycemic group (75% vs. 49%, *p* < 0.0001). CKRT prescribed dose was higher in the euglycemic group than the hyperglycemic group, with a larger percentage of those in the euglycemic group receiving a prescribed dose of > 40 ml/kg/h (65% vs. 53%, *p* = 0.001) (Table 1). There were no statistically significant differences in total CKRT days, CKRT liberation success nor KRT dependence at discharge (Table 2).

**Table 2:** Clinical outcomes between those with euglycemia and hyperglycemia

Characteristic	Euglycemic *N* = 512^1^	Hyperglycemia *N* = 473^1^	*p*-value^2^
Short term			
Mortality at 30 days	96 (26%)	111 (28%)	0.463
Hospital mortality	162 (32%)	210 (44%)	< 0.001
Length of stay^1^	38 (23, 64)	42 (28, 83)	0.017
CKRT days	6 (3, 13)	7 (3, 15)	0.131
Success of initial CKRT liberation	198/347 (57%)	143/286 (50%)	0.084
Long term			
KRT dependance at discharge^1^	57/350 (16%)	43/267 (16%)	0.983
Serum creatinine at hospital discharge^1^	0.46 (0.29, 0.80)	0.46 (0.29, 0.80)	0.973
KRT dependence at 90 days^1^	51/351 (15%)	39/270 (15%)	0.350
Serum creatinine at 90 days^1^	0.40 (0.26, 0.66)	0.46 (0.28, 0.72)	0.130

^1^Among survivors, ^2^ Pearson’s Chi-squared test, Wilcoxon rank sum test

### Outcomes

**Fig. 1:**
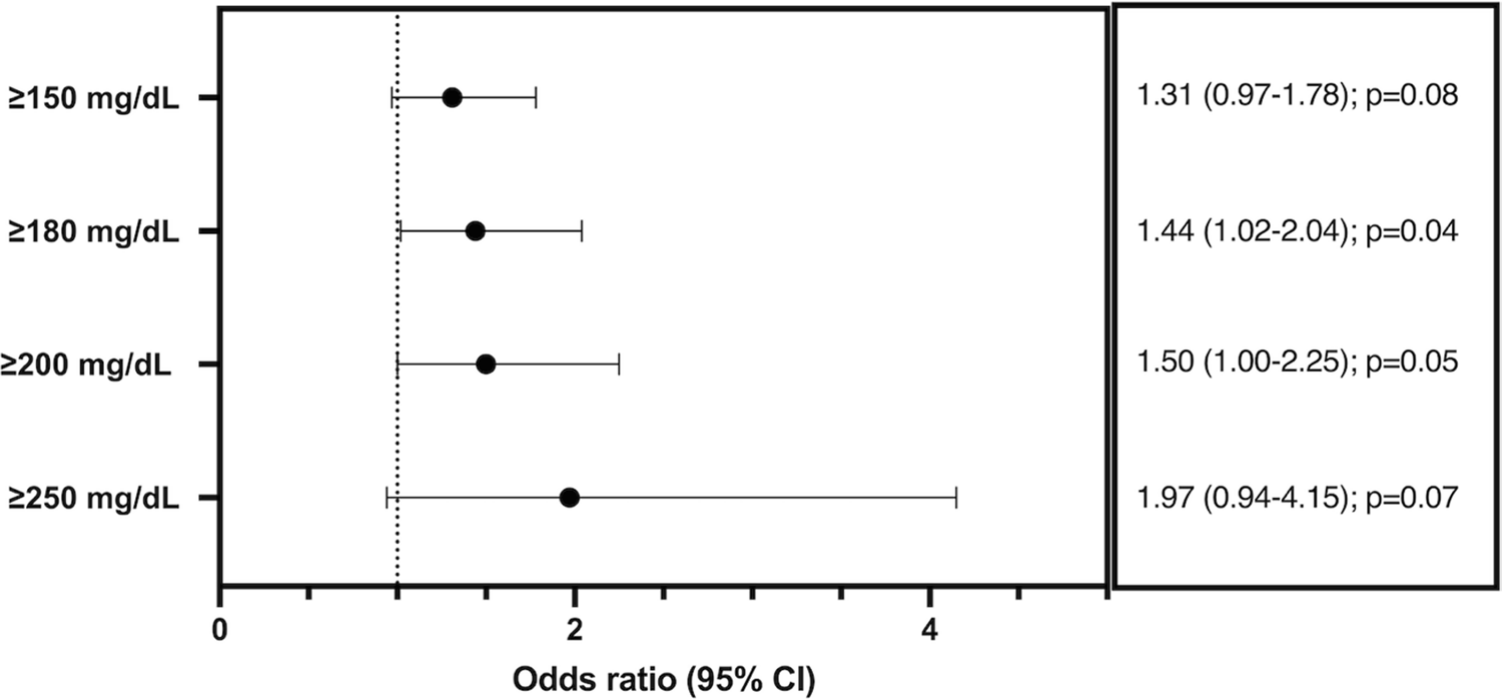
Hyperglycemia and adjusted odds ratio with MAKE- 90 outcomes

MAKE- 90 occurred in 628/985 patients (64%). Of the patients who experienced MAKE- 90, death occurred in 368 (59%). Among the 260 survivors with MAKE- 90, 170 (65%) had > 125% increase in serum creatinine from baseline and 90 (34%) were KRT-dependent (Table 3, Fig. 1). The hyperglycemia group had 326 (68%) with MAKE- 90 occurrence while 302 (58%) in the euglycemia group experienced a MAKE- 90 outcome (*p* = 0.004). The difference in MAKE- 90 outcome was largely due to differences in mortality, as 64% in the hyperglycemic group that had a MAKE- 90 outcome died by 90 days, vs. 53% in the euglycemic group (*p* = 0.006). Persistent kidney dysfunction and KRT dependance at 90 days were not significantly different between the groups (Table 3).

**Table 3:** MAKE- 90 and MAKE- 90 outcomes between those with euglycemia and hyperglycemia

Characteristic	Euglycemic *N* = 512	Hyperglycemic *N* = 473	*p*-value
MAKE- 90 outcome	302/512 (58%)	326/477 (68%)	**0.004**
90-day mortality	160/302 (53%)	208/326 (64%)	**0.006**
Persistent kidney dysfunction			
> 25% decline in eGFR	91/302 (30%)	79/326 (24%)	0.096
KRT dependance at 90 days	51/302 (17%)	39/326(12%)	0.078
MAKE- 30 outcome	198/512 (39%)	215/473 (45%)	0.031
30-day mortality	95/198 (48%)	129/215 (60%)	0.014
Persistent kidney dysfunction			
> 25% decline in eGFR	51/198 (26%)	44/215 (20%)	0.469
KRT dependance at 30 days	52/198 (26%)	42/215 (20%)	0.103

MAKE- 30 occurred in 413/985 patients. The hyperglycemia group had 45% with MAKE- 30 occurrence while 39% of the euglycemia group experienced a MAKE- 30 outcome (*p* = 0.031). The difference in MAKE- 30 outcome was largely due to differences in mortality, as 60% in the hyperglycemic group that had a MAKE- 30 outcome died by 30 days, vs. 48% in the euglycemic group (*p* = 0.014). Persistent kidney dysfunction and KRT dependance at 30 days were not significantly different between the groups (Table 3). In-hospital mortality was higher among the hyperglycemic group (44%) compared to the euglycemic groups (32%, *p* < 0.001) (Table 2). Among survivors, the LOS was longer for the hyperglycemia group compared to the euglycemic group (45 days [IQR 28, 84] vs. 38 days [IQR 23, 64] *p* = 0.009).

In univariate analysis, hyperglycemia (glucose > 150 mg/dL) was associated with MAKE- 90 (OR 1.36, [95% CI 1.02, 1.81]); however, this association did not remain significant in multivariate analysis after adjusting for confounding factors (aOR 1.31 [95% CI 0.97–1.78]). Higher glucose thresholds were significantly associated with MAKE- 90; however, the persistence of this finding after adjustment for age, sex, insulin use, citrate use, presence of sepsis, vasoactive-inotropic score at CKRT initiation and oncologic, immunologic, endocrinologic, or gastrointestinal comorbidities varied by threshold. There were increased odds of MAKE- 90 in those with an average glucose ≥ 180 mg/dL, who had almost 1.5-fold greater odds of MAKE- 90 (aOR 1.44 [95% CI 1.02–2.04]). On unadjusted analysis, those with an average glucose ≥ 200 mg/dL had almost 1.6-fold greater odds of MAKE- 90 (OR 1.60 [95% CI 1.09–2.35]) and those with an average serum glucose ≥ 250 mg/dL had over twofold greater odds of MAKE- 90 outcomes (OR 2.17 [95% CI 1.05–4.46]). However, the association between both higher thresholds did not persist after multivariate adjustment (Fig. 1). When glucose was considered as a continuous exposure variable, each 10 mg/dL increase in serum glucose above 150 mg/dL was associated with a 3.2% increased risk of MAKE- 90 (aOR 1.032 [95% CI 1.0003–1.061]).

## Discussion

In this secondary analysis of the multinational, multicenter WE-ROCK study, hyperglycemia was common in children treated with CKRT for AKI or FO, occurring in half of the cohort. Hyperglycemia as defined by the PODIUM consensus group was not associated with MAKE- 90, but a glucose serum threshold ≥ 180 mg/dL was associated with an exponential increase in MAKE- 90, an association that was largely driven by mortality. We also note differences in the rate of hyperglycemia in children with sepsis and underlying co-morbidities, which may reflect differences in underlying pathophysiology, glucose homeostasis, or other factors such as exposure to glucocorticoids.

Optimal glucose levels in critically ill children and young adults, including those treated with CKRT, remain controversial with inconsistent findings and recommendations based primarily on adult studies [21, 22]. The HALF-PINT trial reported children with tight glycemic control (80–110 mg/dL) had higher rates of CKRT [9]. However, a pediatric meta-analysis reported that tight glucose control was associated with decreased CKRT use (OR 0.63 [95% CI 0.45, 0.86]) [9]. Our findings are consistent with these mixed results as we report that participants with serum glucose ≥ 180 mg/dL have higher odds of poor kidney outcomes; however, this finding did not persist with higher glucose thresholds. The negative impacts of hyperglycemia on kidney function are likely part of a complex interplay of the kidney’s ability to maintain glucose homeostasis and insulin as its main regulator [6]. The kidney contributes to 25% of systemic glucose production and 20% of glucose uptake [23]. Renal oxidative stress is activated and mitochondrial damage increases during hyperglycemia leading to proximal tubular injury [24]. In addition, AKI also contributes to insulin resistance through the accumulation of non-esterified fatty acids resulting in mitochondrial dysfunction subsequently worsening AKI [6, 25].

The use of insulin in children and young adults on CKRT with hyperglycemia has not been studied. While consensus criteria categorize euglycemia as < 150 mg/dL, there is heterogeneity in the glucose thresholds and insulin use across these studies [2, 9, 19, 26]. These varied thresholds contribute to differing conclusions on the association between glucose control and CKRT use. While guidelines on glucose control in critically ill patients exist, there are no clinical guidelines suggesting when insulin should be used [27]. Existing guidelines suggest that most clinicians are more likely to initiate insulin when glucose concentrations consistently surpass 180 mg/dL or even 200 mg/dL [28]. This high threshold may be due to concerns about hypoglycemia with insulin administration and associated morbidity, mortality, and long-term neurocognitive effects. Many studies, including the HALF-PINT trial, were halted due to concerns for hypoglycemia or report rates of hypoglycemia as high as 25% in the tight glucose group [9, 26]. Furthermore, children with AKI are at increased risk of hypoglycemia, as reduced kidney function prolongs the half-life of insulin which can result in vasodilatory effects with orthostatic change; however, this has not been clearly described in trials [6, 29]. We did not capture the lowest glucose concentration across CKRT days and are therefore unable to assess hypoglycemic episodes in our cohort. This is an area that merits future study.

We report higher rates of citrate anticoagulation among the hyperglycemic group. Citrate regional anticoagulation uses dextrose-containing solution (acid citrate dextrose formula A or ACD-A) with 2.45 g of dextrose per 100 mL citrate solution [30]. An adult study of patients treated with continuous veno-venous hemodiafiltration (blood flow of 100 mL/min, 2000 mL/h total clearance) described a glucose uptake of 567 mmol per day with ACD-A anticoagulation contributing to substantial bioenergetic gain [31]. We note that there are differences in anticoagulation choice by center, which may account for some of this finding. Although CKRT results in a profound loss of micronutrients and protein, there is limited knowledge on pediatric CKRT as a source of calories and supply of energy especially glucose, in the form of citrate anticoagulation [32]. We also report lower prescribed dialysis doses among those in the hyperglycemic group; however, the clinical significance of this finding merits further study. Increased awareness of the risk of hyperglycemia in children on CKRT managed with citrate regional anticoagulation is supported by this study. Further studies are needed to better understand the impact of citrate on nutritional status and patient outcomes [33].

The limitations of our study include the retrospective nature and granular glycemic data not being captured by the primary WE-ROCK study. Only a single highest daily glucose was available, which excludes data on any hypoglycemic events, and may not represent the subtlety of glucose changes over a 24-h period. Secondly, we do not have information on glucocorticoid use nor glucose content in dialysis bags, intravenous fluids, nor from parenteral or enteral nutrition. Furthermore, despite our statistical analysis, it is likely that residual confounding based on underlying disease and other factors remains. While we note statistical differences in prescribed CKRT dose between groups, we did not capture data on delivered dose and note the dynamic nature of this data. Finally, the retrospective nature of the study precludes the implication of causality as we are unable to ascribe these associations to the perturbed metabolic milieu in critical illness versus a direct effect of hyperglycemia on kidney function.

In conclusion, we describe the largest pediatric retrospective cohort of CKRT investigating the association between hyperglycemia and kidney outcomes and found worse kidney outcomes and increased rates of death with hyperglycemia in young persons treated for AKI and fluid overload with CKRT. Furthermore, serum glucose > 180 mg/dL was associated with worse kidney outcomes. Hyperglycemia may be a risk factor for poor outcomes in children treated with CKRT and further studies should evaluate preventive and therapeutic measures as delineated by KDIGO [8]. Future studies are needed to focus on short- and long-term kidney outcomes in children with higher glucose thresholds and insulin pharmacokinetic studies are needed to define the optimal range and dose to consider treatment of hyperglycemia to reduce mortality and worse kidney outcomes.

## Supplementary Information

Below is the link to the electronic supplementary material.

Graphical abstract (PPTX 477 KB)

Supplementary file2 (DOCX 32 KB)

Supplementary file3 (DOCX 353 KB)

## Data Availability

De‐identified summary data are available through the WE-ROCK collaborative. Data dictionaries, in addition to study protocol, will be made available upon request. More information about the process and available data can be obtained by contacting the corresponding author (MCS). The data from the WE-ROCK collaborative will be made available to researchers who provide a methodologically sound proposal for use in achieving the goals of the approved proposal following an application process and execution of a data-use agreement as required by the Institutional Review Board at the Cincinnati Children’s Hospital Medical as part of the approval of this collaborative study.
