# Supplementary material for: Hyperglycemia and kidney outcomes in critically ill children and young adults on continuous kidney replacement therapy
Source: Pediatr Nephrol. 2025 Apr 24;40(9):2957–66. doi: 10.1007/s00467-025-06777-3 (PMC12296759; doi:10.1007/s00467-025-06777-3)
Supplement: Supplementary file 1 — Graphical abstract (PPTX 477 KB) [file 467_2025_6777_MOESM1_ESM.pptx]

## Slide 1
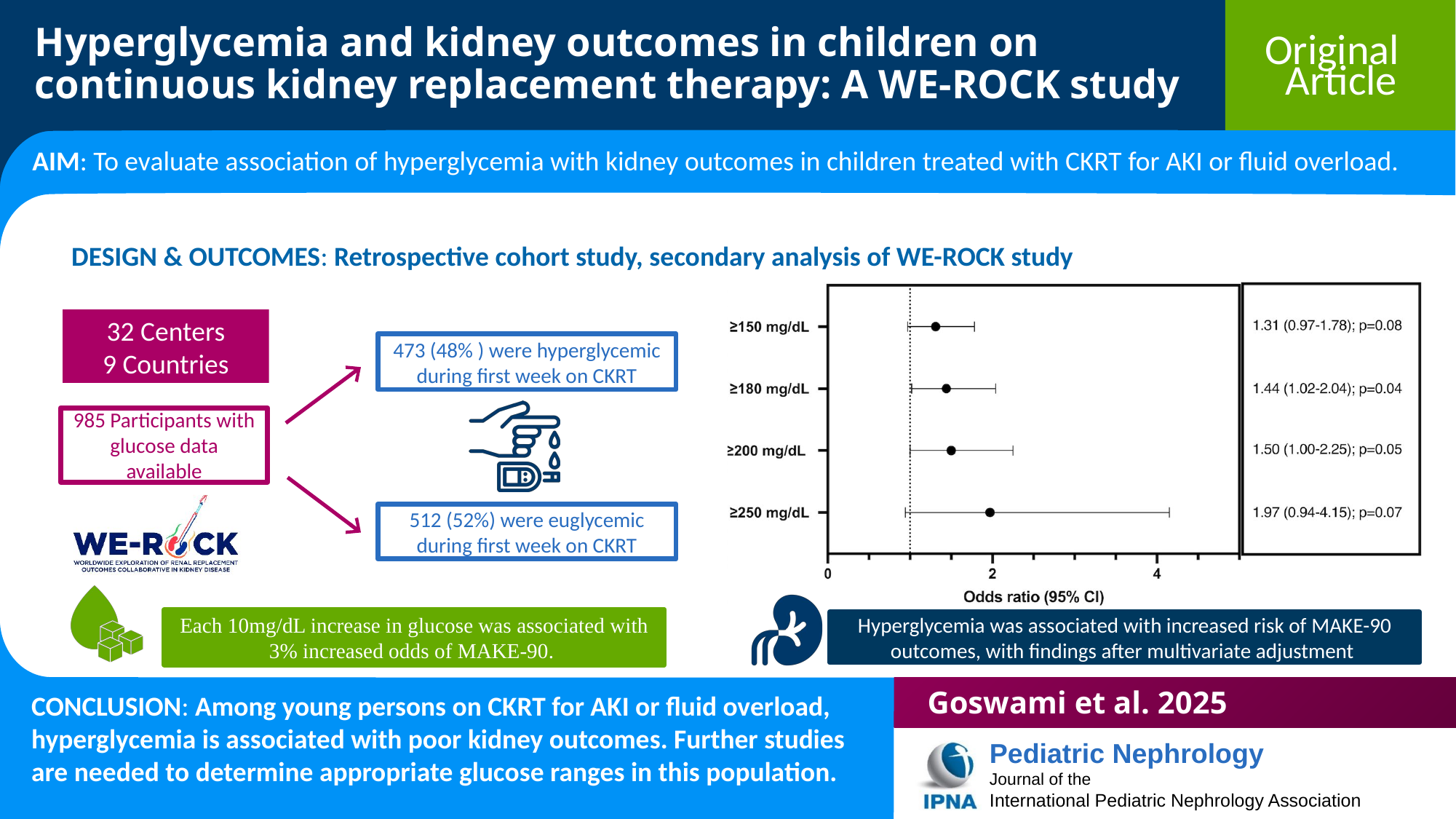

Hyperglycemia and kidney outcomes in children on continuous kidney replacement therapy: A WE-ROCK study
AIM: To evaluate association of hyperglycemia with kidney outcomes in children treated with CKRT for AKI or fluid overload.
DESIGN & OUTCOMES: Retrospective cohort study, secondary analysis of WE-ROCK study
32 Centers
9 Countries
473 (48% ) were hyperglycemic during first week on CKRT
985 Participants with glucose data available
512 (52%) were euglycemic during first week on CKRT
Each 10mg/dL increase in glucose was associated with 3% increased odds of MAKE-90.
Hyperglycemia was associated with increased risk of MAKE-90 outcomes, with findings after multivariate adjustment
Goswami et al. 2025
CONCLUSION: Among young persons on CKRT for AKI or fluid overload, hyperglycemia is associated with poor kidney outcomes. Further studies are needed to determine appropriate glucose ranges in this population.
-----
