# Supplementary material for: Hyperglycemia and kidney outcomes in critically ill children and young adults on continuous kidney replacement therapy
Source: Pediatr Nephrol. 2025 Apr 24;40(9):2957–66. doi: 10.1007/s00467-025-06777-3 (PMC12296759; doi:10.1007/s00467-025-06777-3)
Supplement: Supplementary file 3 — Supplementary file3 (DOCX 353 KB) [file 467_2025_6777_MOESM3_ESM.docx]

Supplemental Figure 1. Consort diagram of those included in the study

**
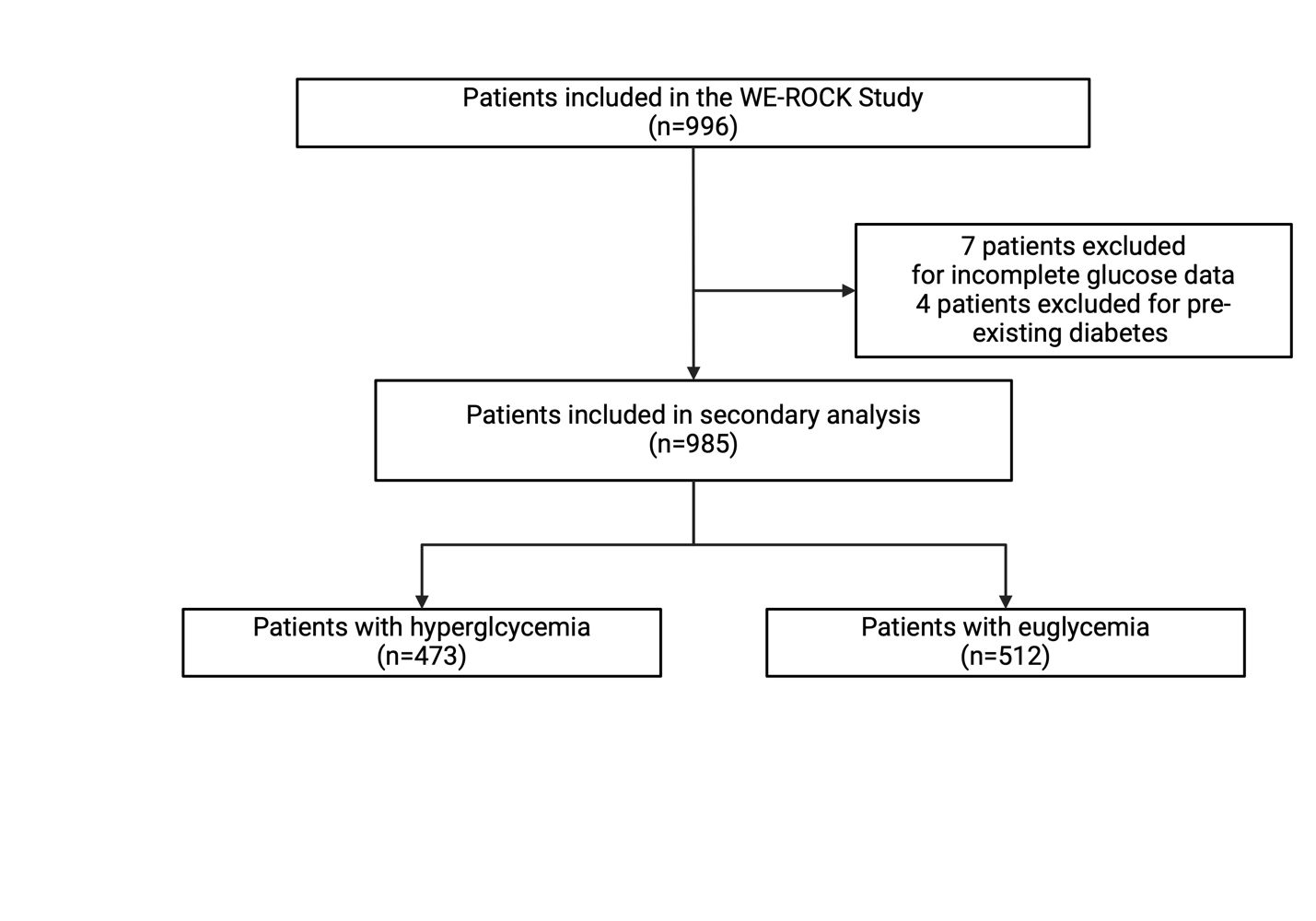
**

Supplemental Figure 2. Distribution of serum glucose values over the day 0 – 7 of those included in the study

**
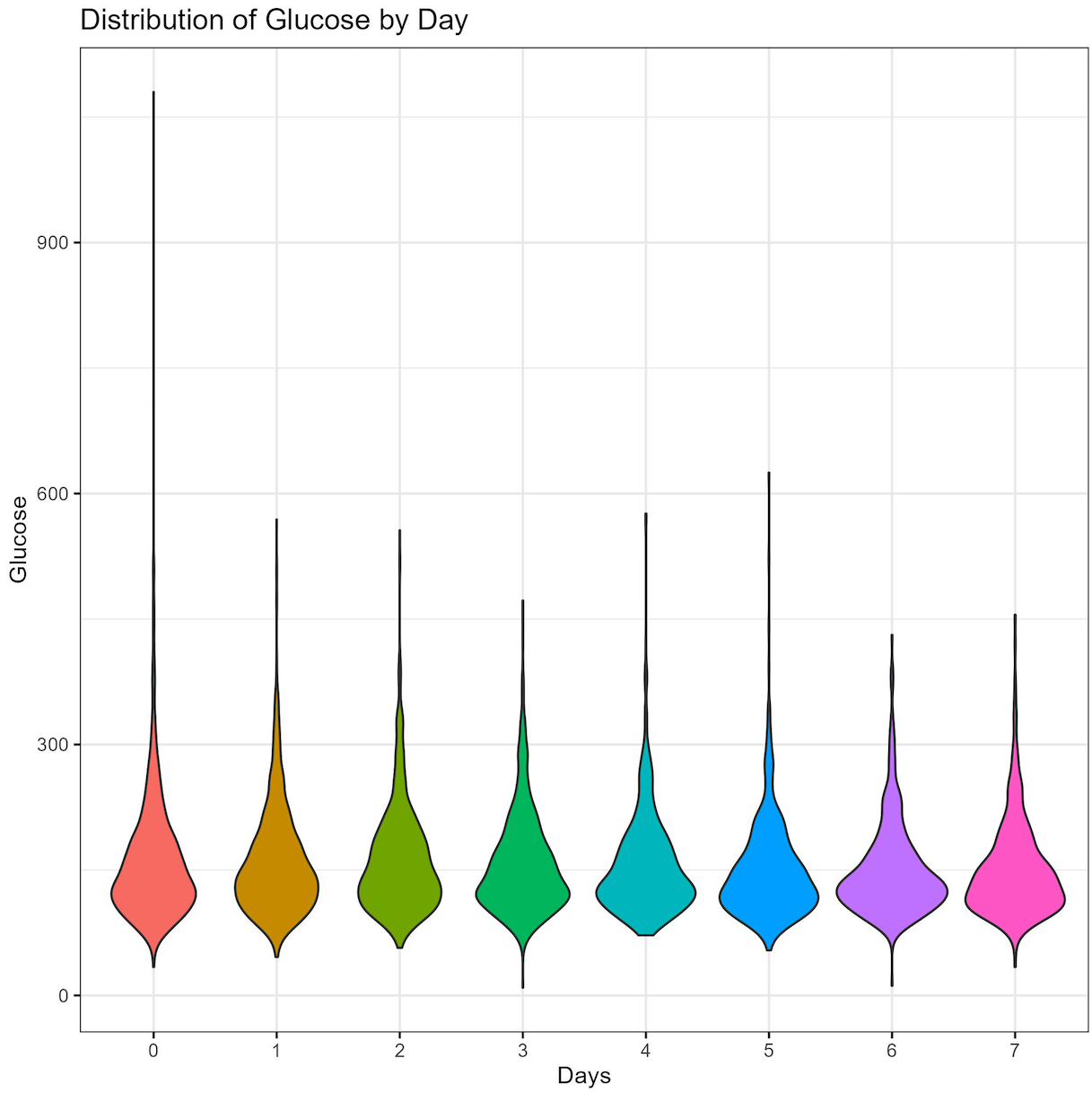
**

**Supplemental Table 1. Association of MAKE90 outcomes within hyperglycemia group with different glucose cut-offs**

| Glucose thresholds | Univariable | | | | Multivariable | | |
| --- | --- | --- | --- | --- | --- | --- | --- |
|  | N | OR*^1^* | 95% CI*^1^* | p-value | aOR*^2^* | 95% CI*^1^* | p-value |
| ≥150mg/dL^3^ | 146/972 | 1.36 | 1.02, 1.81 | **0.035** | 1.31 | 0.97, 1.78 | 0.079 |
| ≥180mg/dL | 74/972 | 1.49 | 1.07, 2.07 | **0.017** | 1.44 | 1.02, 2.04 | 0.040 |
| ≥200mg/dL | 45/972 | 1.60 | 1.09, 2.35 | **0.017** | 1.50 | 1.00, 2.25 | 0.051 |
| ≥250mg/dL | 10/972 | 2.17 | 1.0.5, 4.64 | **0.035** | 1.97 | 0.94, 4.15 | 0.074 |
| *^1^* OR, odds ratio; CI, confidence interval  ^2^ aOR, adjusted odds ratio; Adjusted for age, gender, insulin use, citrate use, presence of sepsis, co-existing morbidities (oncologic, immunologic, gastrointestinal, endocrinologic) and VIS prior to CRRT | | | | | | | |
